# Health System Factors Influencing Partner Notification for STIs and HIV in Lilongwe Malawi. A Pre-intervention Phase Assessment for a Quality Improvement Project

**DOI:** 10.4172/2576-1420.1000125

**Published:** 2018-03-23

**Authors:** Mitch Matoga, Pearson Mmodzi, Cecelia Massa, Agatha Bula, Mina Hosseinipour, Charles Chasela

**Affiliations:** 1 University of North Carolina Project, Lilongwe, Malawi; 2 University of North Carolina, Chapel Hill, North Carolina, USA; 3 University of the Witwatersrand, Johannesburg, South Africa; 4 Right to Care, EQUIP, Centurion, South Africa

**Keywords:** Health system, Partner notification, Sexual partner, HIV

## Abstract

**Introduction:**

Despite its wide use, passive partner notification (PN) has a low yield of sexual partners influenced by patient-related and health system (HS) factors.

**Methods:**

We conducted a qualitative study and clinic observations during a pre-intervention phase of a quality improvement (QI) project to identify HS factors that influenced passive PN at Bwaila STI unit (BSU) in Lilongwe Malawi from January to February 2016. We conducted 15 in-depth interviews with health workers and clinic observations for six clinic flow and PN processes at the clinic.

**Results:**

The majority of health workers felt that the lack of incentives for sexual partners or couples who presented to the clinic was the most important negative HS factor that influenced passive PN. We observed an average clinic start time of 09:02 hours. The average duration of the group health talk was 56 minutes and there was no difference in the time spent at the clinic between index cases and partners (1 hour 41 minutes versus 1 hour 36 minutes respectively).

**Discussion:**

Lack of incentives for sexual partners or couples was the most important HS factors that impacted the yield of sexual partners. Interventions focusing on designing simple non-monetary incentives and QI of passive PN should be encouraged.

## Introduction

Partner notification (PN) was first used around the 19th century in syphilis control but since 1985, it has been used in the control of HIV transmission [1,2]. PN is a process of informing sexual contacts of a patient with HIV/STI that they may be at risk [3]. It is cost-effective in identifying and averting STIs and HIV and it is being used in STI and HIV control programs in many parts of the world [1–5]. The benefits of PN include early diagnosis/screening, early access to treatment and linkage to care, prevention of onward transmission of infection and provision of access to counselling on sexual risk reduction [6].

Despite the several strategies for partner notification, passive PN is the recommended first step in partner notification for resource-limited settings (RLS) by the World Health Organization (WHO) and it is the most widely used and preferred method by both providers and patients in many settings [1–7]. With passive PN, index patients notify their sexual partners on their own and refer or bring the partners to the hospital [5]. Passive PN is the standard of care (SOC) method for PN in Malawi and many other countries within the sub-Saharan African region. Despite being the most preferred and widely used method, passive PN has minimal success in identifying sexual partners (10). In Malawi, a recent study showed that only 24% of partners returned to the clinic for HIV testing and counselling through passive PN (10). A recent national quarterly review reported that PN was conducted in about 30% of index cases [8]. The poor usage and yield of sexual partners through passive PN is unsatisfactory and poses a big challenge to the prevention of HIV and STI transmission.

The yield of sexual partners through PN is either influenced by health system factors or patient factors which arise from social, cultural, ethical and structural factors. These factors may either function as negative influencers (barriers) or positive influencers (facilitators) to the PN process. Patient factors have been studied extensively by other researchers [1,6,7,9–15]. Few health system factors influencing PN have been reported. Health system factors that bar PN include limited resources, poor diagnostic and management structures, work overload for providers, lack of trained staff and unclear ethical guidelines for PN [1,11–15]. Health system factors that facilitate PN are clear clinical and ethical guidelines, legal frameworks around PN, financial incentives, raising awareness on HIV and STIs in the community and training and support for healthcare workers (HCWs) conducting PN [12]. Some of these factors may be common between settings with some setting-specific differences. In Malawi, there is lack of data on health system factors affecting PN at STI clinics. Understanding these factors could help decision makers to identify interventions to best address them. We aimed to identify health system factors that influence PN at Bwaila STI clinic, a busy and dynamic clinic in Lilongwe, the capital city of Malawi. Findings from this study were used to tailor design a quality improvement project with an aim of improving the passive PN process and in turn increase the proportion of sexual partners presenting to the clinic.

## Materials and Methods

We conducted a qualitative study using in-depth key-informant interviews and clinic observations during a pre-intervention phase of a quality improvement project at Bwaila STI unit (BSU) in Lilongwe, Malawi from January to February 2016. BSU is a specialized STI clinic under the Bwaila District Hospital which is a public secondary care facility. BSU is situated in a busy central town area of the Lilongwe district and serves about 80 patients per day. It operates on week days from 7:30 AM to 4:30 PM and receives referrals for complicated STI cases from primary care facilities. The University of North Carolina Project (UNC Project) and the Lighthouse Trust conduct research activities at the clinic and also help provide general care services in collaboration with the district hospital staff. The clinic is usually run by one medical doctor, one clinical officer, four nurses, five HIV testing counsellors and other support staff.

At BSU, patients are first received at the reception for registration of demographic information into the clinic register. After registration, patients are directed to a waiting area where group health talks are conducted by a trained receptionist with the help of a nurse or counsellor. Patients who have a documented HIV test result are directed to the treatment rooms to receive care usually administered by nurses while patients who have never tested or without a documented HIV test result are sent to the HIV testing counsellors for testing. After testing, patients are directed to the treatment rooms to receive care. All patients go back to the reception for final data entry (signs, symptoms, diagnoses and treatment) before exiting the clinic.

We developed a structured interview guide. All HCWs at BSU were eligible and were invited to join the study. There were no exclusion criteria. Fifteen (15) out of 20 HCWs consented for the interviews. The interviews lasted between 15 to 30 minutes. All interviews were conducted in the local language (Chichewa) by the investigator and were recorded. The interviews were transcribed and translated to English by the investigator. Data were analysed using NVivo version 11.0 software. Transcripts were proofread several times by the investigator and all unclear sections were checked and corrected against the audio recordings and field notes. We used a thematic analysis approach to identify emergent codes which were linked to the raw data. The topics which emerged during analysis and the interview guide were used to develop codes and the codes were compared based on frequencies and salience. Coding was done by the investigator and a second reviewer with qualitative research expertise. Codes were compared for congruency and a standby third reviewer was available to resolve coding discrepancies.

We also conducted clinic observations which were guided by the findings from the in-depth interviews. Observations were conducted to better understand the PN process and the general conduct of the clinic (clinic systems). Clinic observations were conducted using a data collection tool by the investigator, a data assistant from UNC project and a receptionist from the district hospital, depending on the process being observed, as detailed in Table 1. Observations were conducted randomly on different days of the week and different times of the day over 1 month in order to obtain results that were representative. Observations were conducted without the knowledge of the staff, except the receptionist and the data assistant, or patients to avoid a Hawthorne effect. We observed the clinic for the following:

- Start time of the clinic/group health talk.
- Duration of the group health talk.
- Proportion of patient who received at least one PN slip to give to their partner(s).
- Duration of waiting time for treatment.
- Total duration of visit and the clinic flow for index cases at the clinic.
- Total duration of visit and clinic flow for partners at the clinic (Table 1).

Clinic start time was defined as the time that the first activity at the clinic (group health talk) started. The waiting time to receive treatment was the duration of the wait after HIV testing to the time patients entered the treatment rooms, while the duration of visits (for index patients or partners) was the total time spent at the clinic from the time of entry to exit.

Data on clinic observations were collected using study-specific data collection forms and entered onto Microsoft Excel spread sheets. We used descriptive statistics such as count, mean, range, and proportion to analyse the data using Microsoft Excel.

Ethical approvals for the study were obtained from the University of the Witwatersrand Human Research Ethics Committee (HREC) and the National Health Sciences Research Ethics committee of Malawi (NHRSC). Permission to use the BSU was obtained from the Lilongwe District Health Office.

## Results

Out of 20 healthcare workers, 15 were interviewed. Five were not available to be interviewed because they were engaged with other duties outside BSU. The median age of respondents was 32 years (IQR: 28, 38). Majority (11/15) of the respondents were female and married (14/15). Among those interviewed, there were 8 HTS counsellors, 5 nurses, 1 receptionist and 1 clinic aide (helper).

### Index patient and sexual partner clinic flow

All the HCWs (15/15) reported that there was no difference in the clinic flow between index cases and sexual partners. They reported that index cases and sexual partners followed the same clinic flow and spent the same duration of time at the clinic.

“Actually, index patients and sexual partners are treated the same way here. They all go through the same steps within the clinic and they spend the almost same amount of time at the clinic.” (ID 12, Female).

### Index cases and sexual partners’ characteristics

Majority (12/15) of the HCWs reported that most of their patients were business people from the busy town area around the clinic. They reported that this was true for both index cases and sexual partners. They also reported that some of the patients they see are not residents of Lilongwe making it difficult to trace them if they are sexual partners.

“Majority of our clients are business people. It is mostly people from the market, bus depot and shops around town-they are vendors, shop keepers, minibus drivers including sex workers. These people do not want to stay in long queues and spend hours here. Also a good number of our clients actually don’t stay in Lilongwe. They come from surrounding districts to the city to conduct business for a short while and return to their homes making them untraceable” (ID 13, Female).

### Facilitators and barriers of PN and pivotal change idea

Few (5/15) of the HCWs reported health system factors that they thought facilitated PN. Among the few who responded, the availability of a television, chairs and a water dispenser in the reception area were the most common factors mentioned.

“I think that the television, the comfortable chairs and water dispenser at the reception area help to entertain patients and make them feel comfortable as they wait to be assisted. This could be a factor that encourages patients to refer or bring their partners to the clinic” (ID 07, Male).

HCWs reported the following health system factors with a barrier effect on PN at BSU.

#### Late start time of clinic activities

Majority (10/15) of the HCWs reported that the clinic usually started its activities late. Due to this, they felt that there was poor organization at the clinic as some patients were sent back home and asked to return the following day. They felt that the lack of organization which resulted in sending back of patients may discourage index patients from referring or bringing their partners to the clinic.

“We see too many patients here, especially after the closure of the STI clinic at Kamuzu Central Hospital. We start the clinic late and usually have few nurses working every day. We also have very few clinic rooms for attending to patients. Due to these factors, we send some patients back home and ask them to come the following morning because we cannot attend to all of them in a day” (ID 02, Female).

#### Lack of incentives for partners and couples

Most (12/15) of the HCWs felt that the fact that sexual partners and couples were not incentivized negatively impacted on PN at the clinic as illustrated below:

“Our clinic does not reward partners or patients who come with their partners to the clinic. Unlike other clinics like the antenatal clinic, women who come with their partners are given preferential treatment and in some cases they are given perks like a maternity start-up pack with a variety of items. I think we need to reward sexual partners and couples in a certain way here.” (ID 03, Female).

#### Lack of space and staff at the clinic

Almost all the key-informants (14/15) alluded to lack of space and staff for efficient conduct of the clinic. They expressed concern that the clinic attended to a lot of patients and that the space and staffing levels available were not adequate to cater for the large patient volumes. They felt that these factors demoralized patients and sexual partners from seeking care at the clinic.

“To be honest, I feel our clinic is too small compared to the number of patients we see. In addition, we also have few staff working here at Bwaila. Even if we were to get more staff, we wouldn’t have enough space to accommodate them” (ID 11, Female).

#### Poor staff attitude

Some key-informants reported that some of the HCWs had poor attitude towards patients. They reported that in some cases patients are shouted at and judged for having an STI.

They felt that such poor attitude discouraged patients or partners from coming to the clinic as expressed below:

“There are some staff, I will not mention their names or positions, who shout at patients like children-especially those who come with recurrent STIs. I do not know if it is due to the large patient volumes or their religious beliefs or whether it’s because they naturally have a bad attitude, but the way they speak to patients sometimes is very inappropriate. There have been cases where some patients, especially females, have come out of some rooms crying.

Do you think that such a patient would come with their partner or refer their partner?” (ID 06, Male).

#### Pivotal change

When asked about what single change idea that HCWs thought would have the greatest impact in increasing the number of sexual partners accessing care at the clinic, majority (11/15) felt that incentivizing partners and couples who come to the clinic would be the best approach. They felt that by doing so, people would be encouraged to refer their sexual partners or come as couples to the clinic. Others reported increasing the staffing levels and space at the clinic.

We observed a total of six clinic flow and partner notification processes. Out of the 10 observations we conducted on clinic start time, the average start time was 09:02 hours. We observed 10 health talks for the duration of health talks and found an average of 56 minutes. All patients (20/20) who were observed for PN slips had at least one PN slip given to them before exiting the clinic. The average duration of waiting time for treatment after HIV testing was 22 minutes.

Total duration of clinic stay for index cases was 1 hour 41 minutes and 1 hour 36 minutes for partners (Table 2).

## Discussion

In a review of health system factors that influenced passive partner notification at a busy and dynamic sexually transmitted infections clinic in a resource-limited setting, we found that lack of incentives for sexual partners or couples who presented to the clinic, inefficient use of available resources and lack of clinic space and staff were the most important health system factors that healthcare workers felt had a great impact on the yield of sexual partners.

Passive PN is a simple and cost-effective intervention that is suitable for RLS for several reasons: (1) HCWs are overworked because of large volumes of patients making provider-oriented PN not ideal; (2) counselling services for passive PN have been easily integrated into clinics; (3) use of counsellors, which is cheaper than using physicians, for counselling on PN has been well accepted; (4) lack of physical addresses, poor road and communication infrastructure and other socio-economic factors make provider-oriented PN difficult to execute in RLS [1].

Within many developing countries, passive PN has a low yield of sexual partners ranging from 20% to 34% [1]. In a 2016 third quarter program review in Malawi, only 13,574 out of 68,989 patients (20%) were sexual partners who presented to the clinic through passive PN [8]. Strengthening of health systems by addressing health system factors affecting passive PN in these settings is one approach for improving the effectiveness of passive PN. However, weak health systems in developing countries are unlikely to emphasize PN when funds for other priorities; essential medicines and other supplies are insufficient [1]. Further, STIs are not perceived as a major public health problem by the policy makers in most developing countries, resulting in inadequate resource allocation [1]. Nonetheless, this is more reason to focus on strengthening health systems to ensure efficient use of the few available resources to enhance PN.

Lack of incentives for partners or couples who presented to the clinic was the most important health system factor that affected passive PN in this study. Majority of the key-informants suggested use of incentives as the best way to improve PN outcomes quickly. Use of incentives in health care has been shown to improve outcomes. Use of unconditional and conditional monetary or non-monetary incentives have been shown to increase HIV testing, increase retention in HIV care, increase condom use and safe sex behaviour, and reduce unwanted pregnancies and HIV acquisition among many other indicators. In Malawi, prioritizing care for pregnant women who came with their spouses coupled with other incentives helped to improve identification of new HIV infections and recruitment of male partners [16–19].

Limited resources, specifically inadequate space and staff and poor management infrastructure due to late start time of the clinic were other common health system factors that affected passive PN at BSU. Healthcare workers at BSU felt that the inadequate space and staff, coupled with late start time of clinic activities, compromised the clinic from running efficiently due to congestion of patients and overload of work as evidenced by the findings from the clinic observations. Health system factors that influence passive PN in RLS are mainly due to limited resources and unclear ethical guidelines legislation and policy for PN [1]. Limited resources include lack of trained staff (especially counsellors) to conduct PN which leads to work overload for the few available HCWs, lack of space, lack of proper diagnostic infrastructure, poor management structures, and lack of essential medication and supplies for treatment [1,15–20]. Redirecting and reordering the few available resources is an effective way of improving the efficiency of health systems and health outcomes [21]. In Canada, researchers used nurses from within an emergency department to specifically attend to non-urgent patients and redirected the flow of non-urgent patients which resulted in a reduction in the average duration of visits.

In our study, poor staff attitude among HCWs at BSU was another important health system factor. The HCWs felt that poor staff attitude was demotivating to index patients and their partners. They feared that the poor attitude may have deterred index cases from referring or bringing their sexual partners to the clinic. In order to achieve efficient PN, high motivation, training on open attitudes and motivational interviewing techniques are needed [22]. This finding suggests the need for periodic training of staff at BSU to keep them motivated, improve their counselling skills and help them attain an unbiased and friendly attitude towards patients.

Lack of PN guidelines/policy was not a contributing health system factor to low PN yield at BSU. Unlike other settings within SSA where PN guidelines/policy do not exist, BSU used guidelines on PN stipulated in the Malawi STI management guidelines for healthcare providers [3]. These guidelines state that; during counselling, attempts should be made by providers to discuss issues surrounding sexual partners; all sexual contacts of persons infected with HIV/STIs should be identified and the PN process should be initiated; the provider should advise and assist patients with identifying the best approach to notify their sexual partners; and PN slips should be provided to patients to give to all their sexual partners [23]. At BSU, all patients received education on PN during a group health talk conducted by a receptionist with the help of a counsellor or nurse. Later patients received counselling on PN individually by nurses when they received clinical care. Patients were given partner notification slips (referral cards) to hand out to their sexual partners as a way of assisting with the PN process. These slips were used to identify patients who presented to the clinic as partners and for administration of appropriate treatment for asymptomatic partners based on the index patients’ STI syndromes.

In another study, clear clinical and ethical guidelines and legal frameworks around PN have been reported as health system factors that facilitate PN [1]. Even though this factor was not explicitly mentioned as a facilitating factor by HCWs at BSU, we feel that it may have contributed positively towards PN as HCWs at BSU were very conversant with the PN guidelines. Other health system facilitators of PN reported in literature include financial incentives, raising awareness on HIV and STIs in the community, and training and support for HCWs conducting PN. At BSU, awareness on HIV and STIs was made through the group health talks and not in the community and the HCWs were adequately trained on the Malawi STI management guidelines for healthcare providers. There were no financial incentives given to patients or their sexual partners at the clinic. However, the availability of comfortable chairs, a water dispenser and a television, which made the clinic visits for patients more comfortable, were some of the factors within the clinic that HCWs felt facilitated PN.

Strength of this study is that it is one of the few studies that investigated health system factors that influence PN in Malawi and SSA. Policy makers and researchers can use these findings to better understand health system impediments to PN in our setting and design feasible and acceptable interventions and policies to improve PN. We used HCWs as experts (key-informants) and custodians of the health system to identify factors. We believe that the expertise from the dynamic range of staff at BSU provided a more representative sample and an accurate assessment of the prevailing health system factors at the clinic. Further, the uniqueness of our study is that it was conducted at a specialized STI clinic. We feel that this setting provided a more specific representation of the factors that affect PN at STI clinics unlike other settings where STI services are integrated with other services.

Conversely, we did not seek patients’ opinions as the consumers of the services of the health system. Possibly, their opinions would have brought a wider and varied perspective. We recommend future research incorporating both heath workers’ and patients’ opinions. Also, specialized STI clinics are associated with stigma and discrimination. The medical fraternity has contributed to the culture of stigma through scare tactics, judgmental attitudes by some HCWs and emphasis on personal autonomy, moral failure and blame [20]. Even though poor attitude among HCWs was one of our findings, HCWs did not allude to issues of stigma or discrimination towards patients. However, this could have been deliberately omitted by HCWs to avoid self-blame. Integration of services, reframing of health campaigns and education of providers to ensure non-judgmental service provision are some of the ways to address stigma inflicted by the health system [20]. We used a receptionist and data assistant at BSU to conduct some of the clinic observations. As part of the clinic staff, they may have introduced some bias in their observations or they may have informed their fellow staff about the observations resulting in a Hawthorne effect. However, we believe that the observations were conducted objectively and over a sustained period of time to counteract this.

## Conclusion

In conclusion, there is need to identify health system factors in order to design interventions with the best-fit to a specific setting. Use of incentives in partner notification should be considered by ministries of health in order to improve the yield of sexual partners through passive partner notification. Furthermore, periodic training of providers conducting partner notification on open, unbiased and friendly attitude and motivational interviewing techniques is warranted in order to keep providers motivated and reduce provider-instigated stigma/discrimination. Also, raising awareness on HIV and STIs and the importance of partner notification in the community is an important public health intervention worth considering. At long-term, effort should be put on increasing the clinic space and the number of staff as one of the ways to improve partner notification. Findings from this research were used to inform a quality improvement project to improve passive partner notification at BSU.

## Figures and Tables

**Table 1:** Details of PN and clinic flow processes observed at BSU.

Observation	Number of observations conducted	Observer
Clinic start time	10	Data assistant Investigator
Duration of the group health talk	10	Data assistant
Waiting time to receive treatment	10	Receptionist Investigator
Proportion of patients who received a PN slip	20	Receptionist
Duration of index patients’ visits	20	Receptionist
Duration of sexual partners’ visits	20	Receptionist

**Table 2:** Results for the baseline clinic observations.

Process Observed	Number of Observations	Findings
Start time of the clinic	10	Average start time: 09:02 hours Range: 08:40-09:45 hours
Duration of health talk	10	Average duration: 56 minutes Range: 20 minutes-1 hour 17 minutes
Duration of wait-time for treatment	10	Average duration: 22 minutes Range: 8 minutes-46 minutes
Proportion of PN slips	20	Proportion: 20/20 (100%)
Duration of clinic visit Index cases	20	Average duration: 1 hour 41 minutes Range: 34 minutes-2 hours 30 minutes
Duration of clinic visit Partners	20	Average duration: 1 hour 36 minutes Range: 32 minutes-2 hours 09 minutes

## References

[1] Alam N, Chamot E, Vermund SH, Streatfield K, Kristensen S (2010). Partner notification for sexually transmitted infections in developing countries: a systematic review. BMC Public Health.

[2] Kissinger PJ, Niccolai LM, Magnus M, Farley TA, Maher JE (2003). Partner Notification for HIV and Syphilis: effects on sexual behaviors and relationship stability. Sex Transm Dis.

[3] Brown LB, Miller WC, Kamanga G, Nyirenda N, Mmodzi P (2011). HIV partner notification is effective and feasible in sub-Saharan Africa: opportunities for HIV treatment and prevention. J Acquir Immune Defic Syndr.

[4] Nichols BE, Götz HM, van Gorp ECM, Verbon A, Rokx C (2015). Partner Notification for Reduction of HIV-1 Transmission and Related Costs among Men Who Have Sex with Men: A Mathematical Modeling Study. Khudyakov YE, editor. PLoS One.

[5] Ferreira A, Young T, Mathews C, Zunza M, Low N, Low N (2013). Strategies for partner notification for sexually transmitted infections, including HIV. Cochrane Database Syst Rev.

[6] Kamanga G, Brown L, Jawati P, Chiwanda D, Nyirenda N (2015). Maximizing HIV partner notification opportunities for index patients and their sexual partners in Malawi. Malawi Med J.

[7] Azariah S (2012). Partner notification for sexually transmitted infections. Why can’t we talk about it?. N Z Med J.

[8] Malawi Ministry of Health (2016). Government of Malawi Ministry of Health Integrated HIV Program Report.

[9] Apoola A, Radcliffe KW, Das S, Robshaw V, Gilleran G (2006). Patient preferences for partner notification. Sex Transm Infect.

[10] Buchsbaum A, Gallo MF, Whiteman MK, Cwiak C, Goedken P (2014). Sexually Transmitted Disease Partner Notification among African-American, Adolescent Women. Infect Dis Obstet Gynecol.

[11] Pavlin NL, Parker RM, Piggin AK, Hopkins CA, Temple-Smith MJ (2010). Better than nothing? Patient-delivered partner therapy and partner notification for chlamydia: the views of Australian general practitioners. BMC Infect Dis.

[12] Reed JL, Huppert JS, Gillespie GL, Taylor RG, Holland CK (2015). Adolescent Patient Preferences Surrounding Partner Notification and Treatment for Sexually Transmitted Infections. Bonsu B, editor. Acad Emerg Med.

[13] Tafuma TA, Ntwayagae BC, Moalafhi CK, Bolebantswe JM (2013). Patient-initiated sexual partner notification in Botswana and time taken for sexual contacts to report for treatment. S Afr Med J.

[14] van Aar F, Schreuder I, van Weert Y, Spijker R, Götz H (2012). Current practices of partner notification among MSM with HIV, gonorrhoea and syphilis in the Netherlands: an urgent need for improvement. BMC Infect Dis.

[15] Wang AL, Peng RR, Tucker JD, Cohen MS, Chen XS (2012). Partner notification uptake for sexually transmitted infections in China: a systematic literature review. Sex Transm Infect.

[16] Bassett IV, Wilson D, Taaffe J, Freedberg KA (2015). Financial incentives to improve progression through the HIV treatment cascade. Curr Opin HIV AIDS.

[17] Solomon SS, Srikrishnan AK, Vasudevan CK, Anand S, Kumar MS (2014). Voucher incentives improve linkage to and retention in care among HIV-infected drug users in Chennai, India. Clin Infect Dis.

[18] Rosenberg M, Pettifor A, Nguyen N, Westreich D, Bor J (2015). Relationship between receipt of a social protection grant for a child and second pregnancy rates among South African women: A cohort study. PLoS One.

[19] Mphonda SM, Rosenberg NE, Kamanga E, Mofolo I, Mwale G (2014). Assessment of Peer-Based and Structural Strategies for Increasing Male Participation in an Antenatal Setting in Lilongwe, Malawi. Afr J Reprod Health.

[20] Morris JL, Lippman SA, Philip S, Bernstein K, Neilands TB (2014). Sexually transmitted infection related stigma and shame among African American male youth: Implications for testing practices, partner notification, and treatment. AIDS Patient Care STDS.

[21] Chartier L, Josephson T, Bates K, Kuipers M (2015). Improving emergency department flow through Rapid Medical Evaluation unit. BMJ Qual Improv reports.

[22] Österlund A (2014). Improved partner notification for genital chlamydia can be achieved by centralisation of the duty to a specially trained team. Int J STD AIDS.

[23] Ministry of Health (2001). Management of sexually transmitted infections guidelines for service providers-Malawi.

